# Using of Essential Oils and Plant Extracts against *Pseudomonas savastanoi* pv. *glycinea* and *Curtobacterium flaccumfaciens* pv. *flaccumfaciens* on Soybean

**DOI:** 10.3390/plants11212989

**Published:** 2022-11-05

**Authors:** Rashit I. Tarakanov, Fevzi S.-U. Dzhalilov

**Affiliations:** Department of Plant Protection, Russian State Agrarian University—Moscow Timiryazev Agricultural Academy, Timiryazevskaya Str. 49, 127434 Moscow, Russia

**Keywords:** soybean, seed treatment, antibacterial activity, bacterial blight, bacterial tan spot, wilt, *Pseudomonas*, *Curtobacterium*, essential oils, plant extracts

## Abstract

The bacteria *Pseudomonas savastanoi* pv. *glycinea* (Coerper, 1919; Gardan et al., 1992) (Psg) and *Curtobacterium flaccumfaciens* pv. *flaccumfaciens* (Hedges 1922) (Cff) are harmful pathogens of soybean (*Glycine max*). Presently, there are several strategies to control these bacteria, and the usage of environmentally friendly approaches is encouraged. In this work, purified essential oils (EOs) from 19 plant species and total aqueous and ethanolic plant extracts (PEs) from 19 plant species were tested *in vitro* to observe their antimicrobial activity against Psg and Cff (by agar diffusion and broth microdilution method). Tested EOs and PEs produced significant bacterial growth inhibition with technologically acceptable MIC and MBC values. Non-phytotoxic concentrations for Chinese cinnamon and Oregano essential oils and leather bergenia ethanolic extract, which previously showed the lowest MBC values, were determined. Testing of these substances with artificial infection of soybean plants has shown that the essential oils of Chinese cinnamon and oregano have the maximum efficiency against Psg and Cff. Treatment of leaves and seeds previously infected with phytopathogens with these essential oils showed that the biological effectiveness of leaf treatments was 80.6–77.5% and 86.9–54.6%, respectively, for Psg and Cff. GC-MS and GC-FID analyzes showed that the major compounds were 5-Methyl-3-methylenedihydro-2(3H)-furanone (20.32%) in leather bergenia ethanolic extract, cinnamaldehyde (84.25%) in Chinese cinnamon essential oil and carvacrol (62.32%) in oregano essential oil.

## 1. Introduction

Soybean (Glycine max Willd) is the main leguminous crop worldwide. Crop is a source of many useful substances [1], and in 2020, 353.5 million tons were harvested in the world [2]. Significant factors in reducing crop yields are weeds, pests and diseases [3,4,5]. Among crop diseases of bacterial etiology, bacterial blight is considered to be the most destructive, reducing yields by up to 40% [6]. The gram-negative bacterium *Pseudomonas savastanoi* pv. *glycinea* (Coerper, 1919; Gardan et al., 1992) (syn—*Pseudomonas syringae* pv. *glycinea* (Coerper, 1919; Young et al., 1978)) (further in the text—Psg) is the causative agent of soybean blight [7]. The disease has been detected in 41 countries covering all climatic zones of soybean production (https://gd.eppo.int/taxon/PSDMGL, accessed on 27 July 2022). Psg affects all aerial parts of the soybean, but the specific symptoms are usually observed on the middle and upper leaves and on the pods. In 5–15 days after infection, necrotic oily spots appear on the leaves surrounded by a chlorotic halo; spots grow and merge, forming necrotic zones [8]. The pathogen is mainly spread through the infected seeds [9] or, more rarely, through the crop residues. The disease reduces yield, soybean oil content, and germination of infected seeds [10].

Another harmful soybean disease of bacterial etiology is bacterial tan spot and wilt caused by a Gram-positive bacterium *Curtobacterium flaccumfaciens* pv. *flaccumfaciens* (Cff) (Hedges 1922). This bacterium affects the vascular system of the plant, causing spots on the leaves, blight, wilting, and death of seedlings and adult plants of leguminous crops [11]. Infected plants grow slowly, their leaves fall off, shoots die off, and the main stem wilts and breaks. Though the dry beans (*Phaseolus vulgaris* L.) are to be the main host plant for Cff, the pathogen can cause outbreaks of disease on soybeans as well [12]. The harmfulness of the pathogen is in reducing the yield [13] and seed quality [14]. Cff has been listed by the European and Mediterranean Plant Protection Organization (EPPO) on the Category A2 List of Quarantine Objects (https://www.eppo.int, accessed on 1 August 2022) (PM1/002 (28) (PM 7/102 (1)). Infected seeds are the main source of infection [15].

Currently, control technology of protecting soybean from bacterial diseases is complex and includes several methods, the main of which is the prevention. In particular, seed certification is the most common method to prevent infected seeds from entering the field [9,11,15]. Other control methods include strict crop rotation, the use of resistant cultivars, and the treatment of seeds and plants with chemical and biological agents. In particular, examples of the use of resistant cultivars are known [10,16,17]; however, the pathogens quickly adapt to them due to the evolution of pathogen virulence and the high diversity of natural populations in general. A radical method of protection is the use of chemical antibacterial substances (in particular, copper compounds and agricultural antibiotics). Unfortunately, their permanent use leads to the development of resistance in bacteria so it is limited in many countries (including Russia), while copper preparations are not effective enough [18], accumulate in plants and soil, and cause environmental problems [19]. There are attempts to use biological agents: antagonistic bacteria [20], PGPR [21], and bacteriophages [22,23] to control the pathogens, but the effectiveness of bioagents depends on the conditions of their use.

On the other hand, the decrease in the number of active substances of fungicides (including bactericides) allowed for use in crop production, concern for the environment, and the development of organic crop production is leading to the development of alternative environmentally friendly pest control systems to combat crop diseases [24]. The use of natural compounds such as essential oils and plant extracts in plant protection against diseases is promising [25,26].

EOs (essential oils) are secondary metabolites derived from various plant parts. In particular, they are reported to be used to control plant diseases of fungal [27], oomycete [28], and bacterial [29] etiology. For example, the mechanism of action of EOs such as thymol (a component of thyme EO) with bacteria is mainly associated with structural and functional changes in the cytoplasmic membrane [30], which leads to damage to the outer and inner membranes; it can also interact with membrane proteins and intracellular targets and affect membrane permeability and lead to the release of K+ and ATP ions [31].

Plant extracts (PEs) such as EOs are composed of secondary metabolites of plant cells but more complex in composition. All of them are biodegradable and do not cause serious harm to the environment. Therefore, EOs and PEs can serve as natural alternatives to pesticides for phytopathogens control [32]. The mechanism of action of PEs mainly consists of the effect on the bacterial cell membrane by changing the internal pH and hyperpolarization of the cell membrane [33].

There are several reports on the determination of the antibacterial activity of EOs and PEs *in vitro* against Psg [6], Cff [34,35], and both bacteria simultaneously [36,37]. A single *in vivo* study of the control of Psg soybean seed infection has been reported [38], while no experiments on Cff infection in soybean have been performed to date. The purpose of this study is to screen the *in vitro* activity of EOs and PEs against soybean bacterial pathogens and evaluate the effectiveness of these substances against an artificial infection on plants.

## 2. Results

### 2.1. Antibacterial In Vitro Activity

The primary antibacterial activity of EOs from 19 plant species and extracts (water and ethanol) from 19 plant species was tested against 3 strains of the *Pseudomonas savastanoi* pv. *glycinea* and 3 strains of the *Curtobacterium flaccumfaciens* pv. *flaccumfaciens* by disc diffusion method.

#### 2.1.1. Antibacterial *In Vitro* Activity by Disc Diffusion Method

Essential Oils. Pathogen susceptibility to essential oils was highly variable and depended on the type of pathogen and source plant (Supplementary Table S1). Zones of bacterial growth inhibition varied from 1.3 mm (peppermint oil against Psg) to 9.3 mm (CCEO against Psg). EOs of Chinese cinnamon and clove showed the highest indices of inhibition zones (9.7 and 9.3 mm, respectively) for Psg. Oregano and thyme EOs showed the highest indices of inhibition zones against Cff (5.7 and 8.3 mm, respectively).

It was discovered that only common rue and tansy EOs did not show activity on any strain of the pathogens. In total, 15 essential oils (78.9%) showed antibacterial activity against Psg and 9 (47.4%) against Cff. Only 7 EOs (36.8%) were active against both bacteria.

Plant Extracts. Susceptibility of bacteria to plant extracts varied and also depended on the type of pathogen and plant (Supplementary Table S1). Zones of inhibition of bacterial growth varied from 1.3 mm (galega extract against Psg) to 6.3 mm (LBEE against Cff). Both against Psg and Cff, LBEE showed the highest indices of inhibition zones (5.3 and 6.3 mm, respectively). It was discovered that activity was shown against at least one species of bacteria for PEs amur cork tree, leather bergenia, cayenne pepper, galega, greater celandine, black mulberry, bridewort, sweet flag, lemon balm, and elderberry. Six extracts showed antibacterial activity on disks (15.8% of 38 extracts in total) against Psg and 8 extracts—against Cff (21.05%). Ethanol extracts were more active than aqueous extracts—8 ethanol (42.1%) and 3 aqueous extracts (15.8%) had an antibacterial effect.

Curiously, the zone of inhibition in the application of the standard antibiotic gentamicin on different strains of Psg and Cff varied. For example, among the Psg strains, the G2 strain is distinguished, which is less susceptible to gentamicin (21.7 mm in diameter), and among the Cff strains, it is F-125-1 with an inhibition zone diameter of 22.7 mm. At the same time, the thiram showed a larger zone of inhibition in Cff than Psg strains (6.3 ± 0.5 mm versus 4.3 ± 0.5 mm), while the antibiotic gentamicin, on the contrary, (Psg 22.7 ± 0.5 < Cff 20.7 ± 0.5).

Subsequently, all EOs and PEs that showed an effect on at least one bacterial strain were used to determine the MIC and MBC values, and G2 for Psg and F-125-1 for Cff were used as target strains.

#### 2.1.2. Antibacterial *In Vitro* Activity by Determination of MIC and MBC Values

**Figure 1:**
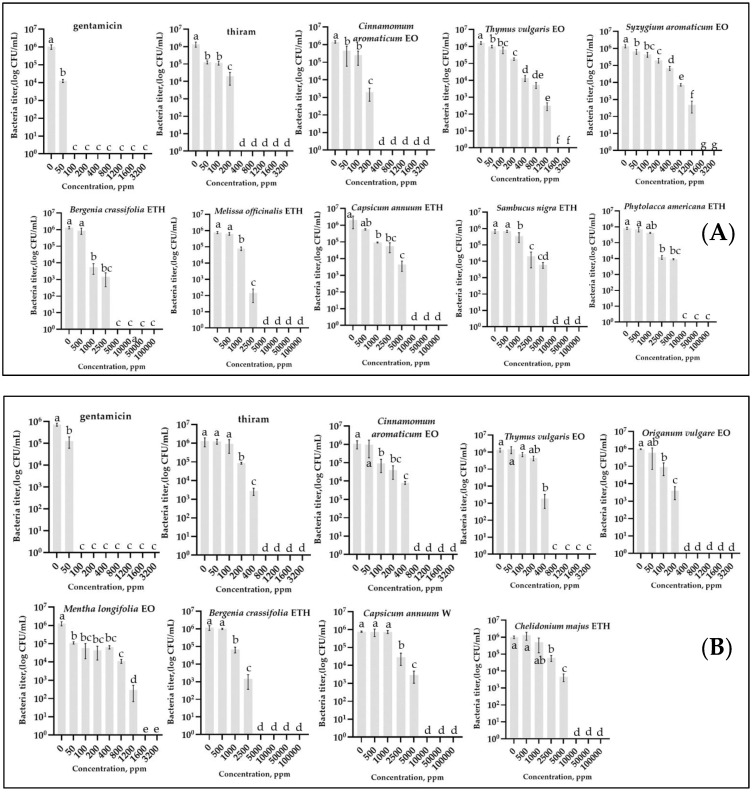
Effect of different concentrations of essential oils and plant extracts on growth of *Pseudomonas savastanoi* pv. *glycinea* strain G2 (**A**) and *Curtobacterium flaccumfaciens* pv. *flaccumfaciens* strain F-125-1 (**B**), measured by counting colonies on agar medium after cultivation in liquid medium. Concentrations are expressed in ppm. EO—essential oil, ETH—ethanolic extract, W—water extract. Values in panels represent the respective mean of two independent trials and error bars represent the standard deviation. Values within columns marked by different letters (a–g) have a significant difference, Duncan’s criteria, *p* = 0.05. A concentration of 0 indicates bacterial growth in the liquid medium without EOs, PEs, standard antibiotics, and thiram. The graphs show only variants with MBC < 1600 ppm for essential oils and <10,000 ppm for plant extracts.

The results of the analysis of bacterial growth, measured by counting the titer after incubation in a broth medium containing various concentrations of EO/PE, are presented in Figure 1 and Table 1. Preliminary experiments showed that the presence of Tween 20 and DMSO in the broth medium in concentrations contained in the tested EOs and PEs did not affect the growth of bacteria. At the same time, only DMSO at high concentrations above 50,000 ppm had a slight negative effect on bacterial growth.

The antibacterial activity of the tested substances is summarized in Table 1, which shows the minimum inhibitory concentration causing growth inhibition (MIC) and the minimum bactericidal concentration (MBC).

Essential Oils. Most of the tested essential oils caused significant inhibition of bacterial growth. The most active EOs with the lowest MIC values were Chinese cinnamon—200 ppm, thyme—800 ppm for Psg, and oregano—200 ppm for Cff. It is worth noting that although the MIC values did not differ for the most efficient EO and thiram for Psg (200 ppm), they were lower for Cff (thiram—400 ppm, oregano—200 ppm). The MBC values for these substances showed a similar pattern. The lowest values were for Chinese cinnamon—280 ppm, thyme—1440 ppm for Psg, and oregano—360 ppm for Cff.

Plant Extracts. The lowest MIC values were for LBEE—1000 ppm and lemon balm—2500 ppm for Psg, and for Cff: LBEE—2500 ppm and cayenne pepper (water)—5000 ppm. The most active EOs, according to MBC values, were the same substances. In particular, for LBEE this indicator was 4000 ppm, for lemon balm—5000 ppm for Psg, Cff: LBEE—5000 ppm and cayenne pepper (water)—9000 ppm. The standard antibiotic gentamicin showed the lowest MIC and MBC values for both bacteria compared to the other treatments (MBC = 80 ppm for Psg and 100 ppm for Cff). Thus, although PEs showed antibacterial activity against the studied bacteria, these concentrations were much higher than those of EOs, the standard antibiotic, and the thiram (~3–15, 20–50, and 5–10 times, respectively).

### 2.2. Phytotoxicity

To assess the determination of the optimal concentrations of EOs and PEs for the treatment of soybean plants, phytotoxicity tests selected 2 EOs (Chinese cinnamon for Psg and oregano for Cff) and 1 PE (LBEE for both bacteria) that showed the lowest MBC values in Section 2.1. A preliminary study showed that the surfactants Tween 20 and DMSO used to dissolve EOs and PEs were only phytotoxic at elevated concentrations. Thus, Tween 20 did not affect seed germination, but caused blight during leaf treatment only at a concentration above 10% in the working solution, while DMSO reduced seed germination at a concentration of 50% and caused blight at a concentration of 20%.

**Figure 2:**
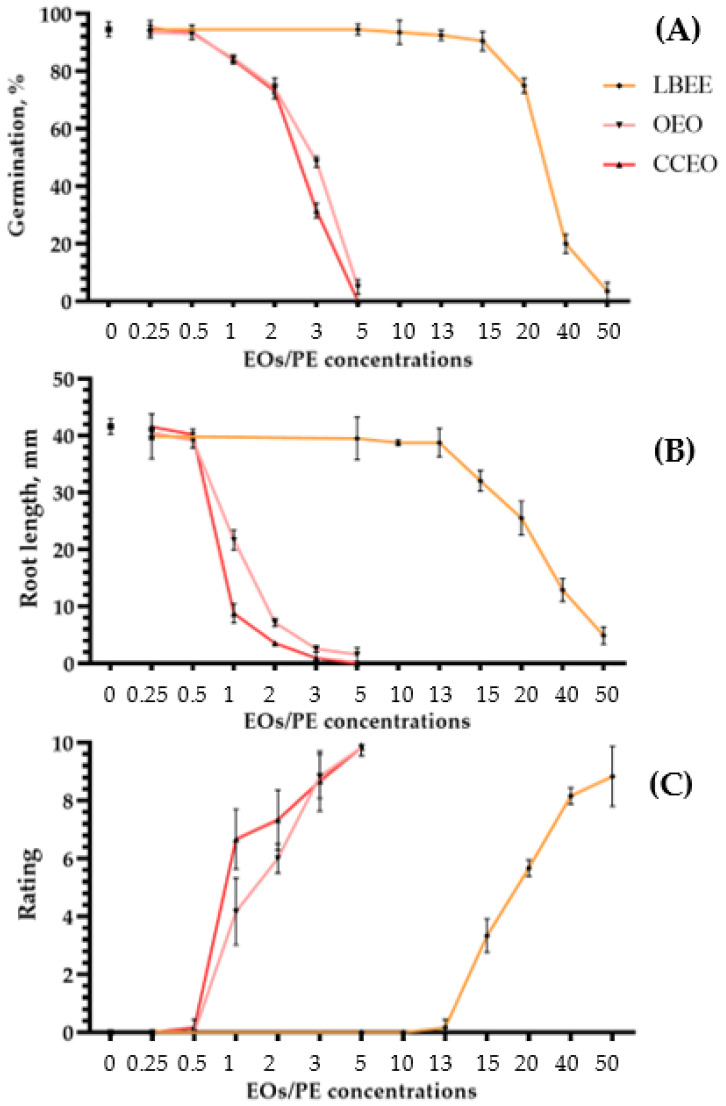
Phytotoxicity of leather bergenia ethanol extract (LBEE), oregano essential oil (OEO) and Chinese cinnamon essential oil (CCEO) on seeds and leaves of soybean. Values of germination (**A**) and root length (**B**) of soybean seeds treated with various concentrations of EOs and LBEE on the 8 DAT. The average score of the integral value of EOs and LBEE phytotoxicity on soybean leaves was 72 h after treatment (**C**). Sterile water was used as a negative control variant. Values in panels represent the respective mean of three independent trials and error bars represent the standard deviation. Values within columns marked by different letters have a significant difference, Duncan’s criteria, *p* = 0.05.

Phytotoxicity on Seeds. The effect of EO and PE concentration gradation on seed germination and soybean seedling root length is presented in Figure 2A,B. Comparing the average values of germination and root length at various concentrations with the control treated with water, we evaluated the phytotoxicity (Supplementary Figure S1).

In both EOs, the threshold of phytotoxic concentrations in seeds was above 0.5%. Although at this concentration a slight decrease in germination and root length is observed for some substances, these values are not statistically significant and do not differ from the control treated with water. In the case of LBEE, a slightly different situation is observed when phytotoxic concentrations start from values above 13%. For example, in the case of germination, a statistically significant decrease occurred only at a concentration of the substance in the working solution of 20% (Figure 2A). The effect of the same extract on the root length of soybean seedlings showed that the phytotoxicity threshold starts from 15% (Figure 2A).

Phytotoxicity on Leaves. Phytotoxicity on soybean leaves was tested by spraying solutions with various concentrations of EOs and PEs. In all cases, the dose-dependent growth of phytotoxicity with an increase in the concentration of the active substance in the solution was determined. Thus, for both EOs, the safe threshold for phytotoxicity, by analogy with seeds, was 0.5% (Figure 2C). Only for CCEO, on single plants, signs of a slight loss of turgor in some leaves were visible, which disappeared in 5–6 days. For LBEE, the maximum concentration of the working solution at which symptoms of phytotoxicity were not visible was also 13% (Figure 2C). The presented threshold values of EOs and PEs concentrations do not statistically significantly differ from water treatment.

### 2.3. Control of Seed and Leaf Infections Psg and Cff with EOs and PE

The relevance and repeatability of models of artificial infections Psg and Cff on soybean plants was described in detail in our previous studies and the experimental conditions were identical to those described earlier in Refs. [22,23].

Efficacy of EOs and PE against Psg and Cff leaf infection. Soybean leaves infected with Psg and Cff were treated with EOs and PE in triplicate. The spread of the disease on the leaves was measured using the Leaf Doctor program 12 days after the treatment of previously infected plants (Supplementary Figure S5).

**Figure 3:**
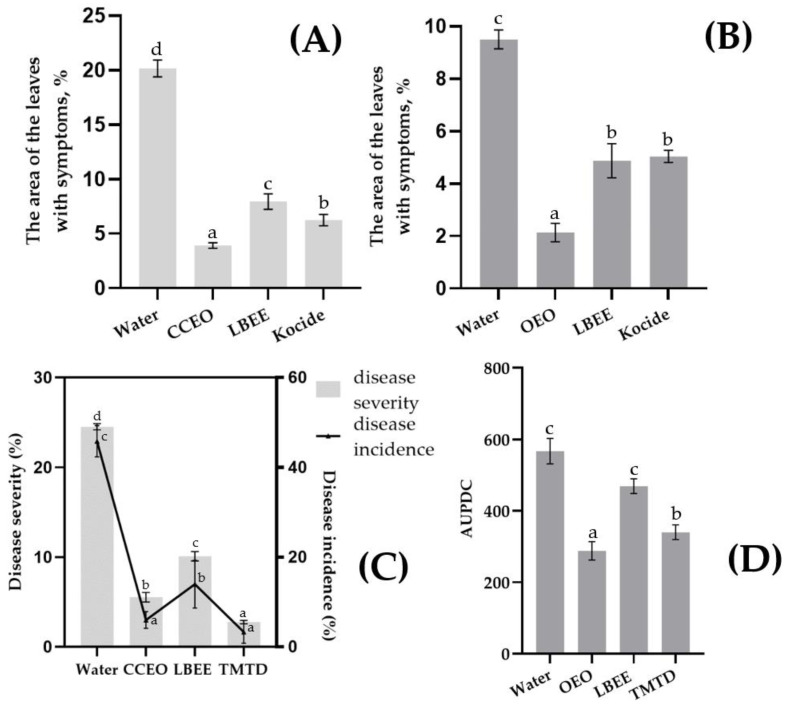
Bacterial blight (**A**,**C**) and bacterial tan spot and wilting (**B**,**D**) of soybean caused by artificial inoculation of Psg and Cff under EOs and PE treatment. Values in panels represent the respective mean of three independent trials and error bars represent the standard deviation. Values within columns marked by different letters have a significant difference, Duncan’s criteria, *p* = 0.05. (**A**): disease severity (diseased leaf area %) on green plants inoculated by Psg or (**B**) inoculated by Cff; (**C**): disease severity (diseased leaf area %) (Left vertical scale, Bars) and disease incidence, % (right vertical scale, line), after soybean seed inoculation by Psg; (**D**): values of AUPDC after soybean seed inoculation by Cff.

In the Psg experiment, disease progression was reduced by 60–80% with variant treatment compared to the water-treated control (Figure 3A). Interestingly, the highest efficiency was observed with the CCEO treatment (80.6%), while the LBEE treatment was inferior (60.5%) to both the CCEO treatment and the standard fungicide Kocide (69.05% efficiency). In the control variant of the experiment with Cff, the average leaf area with symptoms of the disease, although inferior to Psg, was at a high level (9.5% and 20.15%, respectively). The development of the disease on the treated variants was reduced by 47.0–77.5% compared to the control treated with water (Figure 3B). The highest efficiency was observed with OEO treatment (77.5%), while LBEE and Kocide showed approximately the same efficiency (48.8 and 47.0%, respectively).

Efficacy of EOs and PE against Psg and Cff Seed Infection. Treatment of soybean seeds previously artificially infected with Psg with experimental variants showed a significant reduction in the frequency of infection of seedlings and the rate of disease development. The control treatment (using water) showed a rapid development of the disease in the plants (Figure 3C). Due to the daily overhead watering of the plants, a secondary infection was observed with a severity similar to the disease outbreak in the field. The biological effectiveness of CCEO treatment was 77.4% (incidence of disease) or 86.9% (severity of disease) compared with control, while LBEE treatment reduced the development and prevalence of the disease by more than 2 times, but was inferior to CCEO treatment. Treatment by thiram greatly reduced both development (88.7% efficacy) and prevalence (92.8% efficacy) of the disease in the trial.

In the control variant with Cff seed infection, symptoms of wilt and yellowing of soybean leaves were observed with average AUPDC = 633 points (Figure 3D). In general, the effectiveness of treatments with experimental variants was lower than in the experiment with Psg. Thus, the biological efficiency of the OEO treatment was 54.6% compared with the control, while the LBEE treatment reduced the AUPDC by only 25.9%. Treatment of seeds with thiram also did not show a high biological effect, displaying the efficiency of 46.3%.

### 2.4. Identification of Chemicals Comprising EOs u PEs

The extract yield from leather bergenia was 4.22% and from OEO 1.69% of the mass of air-dry plants.

GC-MS and GC-FID analysis of CCEO, OEO, and LBEE identified 58 compounds (Table 2). Twenty-two compounds from LBEE, 18 from CCEO, and 24 from OEO were identified, representing 90.63%, 99.68%, and 99.49% of identified compounds, respectively, for each EO/PE. In LBEE, the most common compounds were acetic acid (27.85%), 5-Methyl-3-methylenedihydro-2(3H)-furanone (20.32%), and eugenol (10.94%) (Supplementary Figure S2). The most common CCEO compounds were cinnamaldehyde (84.25%), cinnamaldehyde dimethyl acetal (3.36%), and o-Methoxycinnamaldehyde (6.91%) (Supplementary Figure S3), while those of OEO were carvacrol (62.32%), cymene (19.85%), and thymol (3.52%) (Supplementary Figure S4).

## 3. Discussion

Bacterial diseases of the soybean are a problem all over the world: among them, Psg is a species that has been causing harm for a long time, while Cff has only recently begun to be registered as a significant crop pathogen [12,39]. At the same time, it is known that both bacteria can form a single pathocomplex and simultaneously infect soybean in the field [40]. The particular danger of both bacteria for soybean cultivation is the main method of transmission—seeds, through which pathogens are transmitted and spread to new locations [9,15].

Currently, there are several strategies for the control of bacterial plant diseases. By analogy with human bacterial diseases, antibiotics should be used, but this is prohibited by the legislation of many countries, including Russia and the EU. Copper-containing fungicides often used to control phytopathogenic bacteria are increasingly excluded (or restricted) from plant protection systems due to legal prohibitions and environmental concerns. Therefore, in areas where diseases are spread, control methods should include the prevention and control of infected seeds using eco-friendly pest control systems [41].

Compared to chemical bactericides and antibiotics, EOs and PEs have many advantages because they are environmentally friendly, have low toxicity to mammals, and are biodegradable in the field when released into the soil [42]. In addition, they seem to be a potential alternative to synthetic substances, in particular, due to the increasingly developing resistance even to multisite biocides in pathogenic microorganisms [19]. In recent years, many studies have been reported describing the strong antibacterial activity of EOs and PEs. Although the bulk of research concerns human pathogens, precedents are known for the use of these substances to combat pathogens that lead to food spoilage [43] or fungi and bacteria that infect agricultural plants [44,45]. Much attention is paid to the use of EOs and PEs as disinfectants for seed disinfection against bacterial and fungal diseases [46,47]. In terms of bacteria, EOs are known to have an anti-quorum sensing effect in addition to direct contact biocidal action, which consists of blocking cell communication, which plays an important role in biofilm development and affects resistance and virulence [48,49]. Moreover, many countries use commercial pesticides based on EOs and PEs. For example, Koppert BioSystems (Veilingweg, The Netherlands) supplies the fungicide Nopas (a.i. EOs thyme and peppermint) to protect tomatoes from root rot; in the USA, fungicides VertigoTM with a.i. cinnamaldehyde [50] and Qwel [51] are used to protect crops against a wide range of pathogens.

In this context, this study aimed to screen for the activity of a range of EOs and PEs against soybean bacterial pathogens and to fill some of the gaps in knowledge about the main aspects of the control of these diseases with botanical pesticides. Most of the compounds tested showed dose-dependent *in vitro* antibacterial activity against both Psg and Cff. These data confirmed previous results obtained for Psg and Cff. In particular, the work [38] reported on the *in vivo* use of thyme EO for the control of Psg seed infection and showed that when treated with this substance, the number of bacteria on seeds decreased by 6%, and germination increased by 21%. The results for Cff are also consistent with those of Flores et al. [52], in which EOs of oregano, thyme, and cinnamon were tested against *Clavibacter michiganensis* subsp. *michiganensis* and oregano EO showed the highest degree of bacterial inhibition. This may be because the genera *Curtobacterium* and *Clavibacter* are closely related and were previously formerly classified as genus *Corynebacterium* sp. [53]. The work [6] reports the antibacterial activity of aqueous extracts of neem (*Azadirachta indica*) and ginger (*Zingiber officinale*), and the work [37] reports the action of carvacrol against Psg strains *in vitro*. Against Cff, EOs of cumin (*Carum carvi*) [36], moshkoorak (*Oliveria decumbens*), and spartan oregano (*Origanum minutiflorum*) [34] exerted strong antimicrobial activity *in vitro*. As far as extracts are concerned, the ethanolic extract of St. John’s wort (*Hypericum perforatum*) displays antibacterial activity against Psg *in vitro*. Given that EOs and PEs are composed of many different secondary metabolites, it is of interest to characterize individual substances with antibacterial activity. In particular, the antimicrobial efficacy of CCEO is due to high phenolic compounds such as cinnamaldehyde and eugenol [54]. In [55], it is reported that the antibacterial activity of OEO is due to the two main phenols carvacrol and thymol. The activity of extracts is also due to different groups of phenolic compounds [56,57].

In this study, the most abundant compound in CCEO was cinnamaldehyde, which corresponds to the literature data [54]. In OEO, the most common compounds were carvacrol and thymol, which also corresponds to the literature data; however, cymene (19.85%) is represented in a smaller proportion than carvacrol, but bigger than thymol, which is of interest given that this compound also has an antibacterial effect [58]. The analysis showed that the predominant compound in LBEE is 5-Methyl-3-methylenedihydro-2(3H)-furanone, the biological effect of which has not yet been published. Other major components of the extract are eugenol and acetic acids, which display antibacterial activity against a wide range of bacteria [59,60].

If at the initial stage all EOs and PEs were tested, then, at the stage of the application on plants, studies were carried out using 3 substances that showed the lowest MBC value: CCEO for Psg, OEO for Cff, and LBEE for both bacteria. Potential phytotoxic effects on soybean were assessed for seeds using a germination test after dip treatment, and for leaves by spraying with a hand sprayer. Considering that a large number of diverse methods used to determine the phytotoxic effects of EOs and PEs concerning plants [61,62] rather complicate the overall picture, it was decided to determine the phytotoxic concentrations for each of the substances planned for use *in vivo* in relation to soybean. Moreover, there are precedents for the use of EOs as contact herbicides for weed control, in which a decrease in crop yield was observed [63,64]. In the current study, all tested substances were phytotoxic to soybean seeds and leaves at certain concentrations. Phytotoxicity analysis of EOs showed that the lowest concentration at which no phytotoxic effect was observed was 0.5%. For LBEE, this figure was 13%. It is known from the literature that the analyzed EOs and PEs have a phytotoxic effect: in particular, OEO against germinating seeds of radish (*Raphanus sativus*) [65], wheat (*Triticum aestivum*) [66], and tomato (*Solanum lycopersicum*) [67]. The work [63] reported on the phytotoxic effect of CCEO in the treatment of apple leaves. Moreover, CCEO has elicitor activity in plants against phytopathogens [68,69]. From the component composition of essential oils, it is known that monoterpenes, in particular 1,8-cineole and carvone, have the greatest phytotoxic effect [70]. Arbutin from *Bergenia crassifolia* [71] and methanolic extract of *Bergenia ciliata* [72] are known to have plant growth-inhibiting properties. In addition, plants from Bergenia species or their specific components can be used as a natural insecticide [73].

Tests on an artificial infection Psg and Cff showed that the analyzing substances can reduce the development of the disease both in the treatment of seeds and in the treatment of soybean leaves. In general, the effectiveness of variants was higher in the control Psg than against Cff. Possibly, this phenomenon is related to the fact that Cff can penetrate the conducting system of the seedling [74] and be inaccessible to contact bactericides, while Psg does not have this ability. The high efficiency of seed treatment with EOs is possibly due to both the treatment method (soaking), in which the antibacterial agent penetrates the thickness of the seed much better than with the traditional semi-dry treatment method, and the ability to evaporate essential oils into a gaseous form, the penetrating ability of which is at or below higher than the liquid form [75].

Undoubtedly, the use of these substances in industrial volumes to control soybean bacterial diseases requires several additional studies. In particular, it is necessary to choose formulation to obtain stable emulsions/solutions of EOs and PEs when treating plants [76,77]. Packaging is also promising, in particular that of EOs in nanoformulations, which make it possible to reduce the consumption rate of the active substance and increase efficiency due to more uniform contact of the substance with the pathogen/plant [78,79]. Evaluation of the individual components of EOs and PEs as antibacterial substances to combat bacterial plant diseases arouses great interest, too. For example, the Chinese cinnamon EO main active ingredient cinnamaldehyde is used as a common commercial pesticide [50].

Thus, the results of this study confirmed the antimicrobial activity of EOs and PEs and showed promising prospects for their potential use also in the treatment of soybean seeds and leaves for protection against bacterial diseases. Because this is an initial report, more research is needed to improve soybean processing efficiency by optimizing delivery technology, plant application, and formulation for commercial use in field conditions.

## 4. Materials and Methods

### 4.1. Bacterial Strains

The work used strains of *Pseudomonas savastanoi* pv. *glycinea* CFBP 2214 and *Curtobacterium flaccumfaciens* pv. *flaccumfaciens* CFBP 3418 from the CFBP collection (Beaucouzé, France) and Russian strains isolated (from damaged by diseases soybean plants in 2019–2021 years) and described by us (Psg: G2 and G17, Cff: F-125-1 and F-30-1) in previous publications [22,80]. These strains were pathogenic against soybean plants cv. Kasatka by artificial infection. Psg strains reacted positively with PCR assay for gene *cfl* [8] and had sequences of gene *cts* fragments [81] that were most similar to the corresponding sequence in the genome of the Psg strains in Genbank. Strains Cff were attributed by PCR using genus-specific [82] and species-specific [83] primers.

### 4.2. Plant Material

The plant samples for the isolation of antibacterial substances were collected during June–August 2021 on the territory of the botanical garden of First Moscow State Medical University (Moscow, Russia) (all excepting a few species), Field experimental station of Russian State Agrarian University (Moscow, Russia) (garlic, cv. Novosibirskiy) and local markets (key lime, mandarin, paprika (country of origin—India)). Chinese cinnamon EO (CCEO) was kindly provided by «SoyuzSnab» company (Krasnogorsk, Russia). The selection of plant species and the EO and/or extracts used was based on preliminary reports (references) of antibacterial activity (Table 3). In two plants (sweet-flag and garlic), both essential oil and extracts were used separately. A complete list of plants and parts from which EOs and PEs were isolated is presented in Table 3. Biological species were identified jointly with specialists from the Botanical Garden following morphological characters and keys that serve as the main basis for the taxonomy of the respective plant families [84].

#### 4.2.1. Extraction of Essential Oils

After harvesting, the plants were dried out of the sun and under natural ventilation for 2 weeks, after which they were cut into small pieces 5–6 mm in size with scissors and subjected to hydrodistillation according to [108] with minor changes. For this, 100 g of each plant was soaked in 2 L flasks with 1500 mL of water, hydrodistilled for 3 h in a Clevenger apparatus, and the collected distillates were dried over anhydrous Na_2_SO_4_. EOs were stored in sealed tubes at 4 °C until analysis.

#### 4.2.2. Extraction of Plant Extracts

Plant samples were preliminarily collected in the same way as in Section 4.2.1. An extraction was carried out in a Soxhlet apparatus according to [109]. For this, 50 g of each plant were crushed in a laboratory mill to a powder, 300 mL of water or 96% ethanol was added, and extracted for 12 h. The resulting solutions were filtered through Whatman No. 1 paper, evaporated, and concentrated to dryness using a RE100-Pro rotary evaporator (DLab, Beijing, China) at 50 °C. The resulting extracts were dissolved in a 4% aqueous solution of DMSO (dimethyl sulfoxide) to a final concentration of 50% (by a.i.) according to [110] with changes and stored in sealed test tubes at 4 °C until analysis.

### 4.3. Determination of Antibacterial Activity

#### 4.3.1. Determination of Antibacterial Activity by Disc Diffusion Method

Substances were screened for antibacterial activity by the disk diffusion method, which is usually used as a preliminary check and for the selection of the effective substances [111] with some changes on all 6 strains. It was performed using 48 h cultures grown at 28 °C on a King B agar medium. The suspension was adjusted to 10^5^ CFU/mL with sterile saline. Then, 100 µL of the suspension was dispensed into plates containing King B using a sterile loop. Discs of Whatmann filter paper (No. 1) with a diameter of 6 mm were cut by punching paper, and disc blanks were sterilized in a hot air oven at 160 °C for one hour.

Essential oils were dissolved in a 2.5% aqueous solution of Tween 20 to a concentration of 5% on a vortex until a stable emulsion was formed. Under aseptic conditions, sterile discs were impregnated with 10 µL of an emulsion (0.5 µL of a.i./disc) of the appropriate essential oils and placed on the agar medium. Plant extracts pre-dissolved in DMSO were tested similarly to essential oils, except that 10 µL of a 50% aqueous/ethanolic extract dissolved in DMSO was placed on the disks.

Discs containing 2.5% aqueous Tween 20 and discs containing 10 µL 4% aqueous DMSO were used as negative controls. Discs containing 0.5 mg (in a.i.) of the antibiotic gentamicin (DalKhimPharm, Khabarovsk, Russia) and reference pesticide thiram (TMTD fungicide, WSC (400 g/L a.i.), Avgust LLC, Moscow, Russia) were used as positive controls). All dishes were sealed with laboratory film to avoid possible evaporation of the test samples. The dishes were left for 30 min at room temperature to allow diffusion of the oil, and then they were incubated at 28 °C for 48 h. After the incubation period, the zone of inhibition was measured with a caliper. The diameters of the inhibition zones of bacterial growth were measured excluding the disc diameters (6 mm). Studies were performed in triplicate of 3 plates with one disk of the defined EO, PE, or standard antibiotic/thiram each, and the mean bacterial growth inhibition zone was calculated.

#### 4.3.2. Determination of MIC and MBC

The activity of EOs and PEs was assessed with Psg and Cff according to the CLSI 2015 broth microdilution method [112] with modifications. Starter cultures were prepared by suspending bacterial cells in 5 mL King B (without agar) and incubating at 28 °C for 24 h at 150 rpm in an ES-20 shaker (BioSan, Riga, Latvia).

Bacterial suspensions were diluted to obtain an absorbance value on a spectrophotometer corresponding to a concentration of 10^5^ CFU/mL. Sterile 1.5 mL Eppendorf-type tubes were filled with liquid King B medium, emulsions or solutions of EO/PE/standard antibiotic/thiram to a predetermined concentration, and 50 µL of a suspension of bacteria strains (Psg G2 and Cff F-125-1). The total volume of the reaction mixture was 1000 µL.

The concentration series for EOs and standard antibiotic/thiram were: 50, 100, 200, 400, 800, 1200, 1600, and 3200 ppm; for PEs: 500, 1000, 2500, 5000, 10,000, 50,000, and 100,000 ppm (in a.i.). After preparing the reaction mixtures, the tubes were incubated at 28 °C for 48 h and 350 rpm in ThermoMixer F 2.0 (Eppendorf, Hamburg, Germany). After 48 h of incubation, 100 µL of the reaction mixture was taken from each tube, a series of tenfold dilutions in sterile water was performed, placed in Petri dishes on King B medium, and spread over the entire surface of the medium using an L-shaped spatula. The dishes were placed in a thermostat at 28 °C for 48 h, after which the concentration of bacteria in each original tube was calculated. The repetition of the experiment was 2 with 3 dishes for each dilution. The minimum inhibitory concentration (MIC) was defined as the lowest concentration of test compounds that caused 90% growth inhibition compared to control, which was determined by calculating the inhibition of bacterial growth. The minimum bactericidal concentration (MBC) was defined as the lowest concentration of an analyte that caused 99.9% death of a bacterium. For a more accurate result, the same experiment was carried out, but with a decrease in the concentration of the analyte to MIC at regular intervals of concentrations (5 points of concentration from the expected MBC to MIC). For further purposes, 2 EOs and 1 PE showing the lowest MBC were used: CCEO for Psg, OEO for Cff, and LBEE for both bacteria.

### 4.4. Phytotoxicity on Soybean Seeds and Plants

The phytotoxicity of EOs and LBEE tested on soybean seeds was assessed by a germination test using the standard “over paper” method described in the International Rules for Seed Testing [113]. Soybean (cv. Kasatka) seeds were treated by immersion in an aqueous solution of EOs or PEs at various concentrations for 10 min and then completely dried on sterile filter paper at room temperature under sterile laminar box conditions. EOs were dissolved in a 2.5% aqueous solution of Tween 20 to a concentration of 5% on a vortex until a stable emulsion was formed and PE was pre-dissolved in DMSO. EOs solutions were diluted to concentrations of 0.25, 0.5, 1, 2, 3, and 5%, LBEE to 2.5, 5, 10, 13, 15, 20, 40, and 50% with sterile water. Seeds soaked in water were used as a negative control. Then, the seeds were kept under conditions of constant humidity and incubated at 25 °C.

Germination was assessed after 8 days; a seed was considered germinated if it produced a sprout with a well-developed root. The average percentage of germinating seeds was determined for all repetitions. After calculating the germination, the cotyledons were separated and the length of the roots was measured using a caliper. The experiment included 3 repetitions of 50 seeds for each variant.

To test the phytotoxicity of substances on vegetative plants, soybeans were grown to phase R1 in a turf–perlite mixture (Vieltorf, Velikiye Luki, Russia) in plastic trays for plant cultivation (volume 1 L, AgrofloraPack, Vologda, Russia).

Plants were kept in a greenhouse at 28/22 °C (14 h day/10 h night) under natural light and watered as needed. After preparation and homogenization, the solutions were applied to plants using a sprayer (with a drop size of ~300 µm) at a working solution consumption rate of ~5 mL/plant (until all leaves were completely wetted).

After treatment, the plants were kept in a greenhouse under the same conditions for 7 days, and then they were evaluated using the phytotoxicity scale [114] where: 0—no symptoms; 1—very slight discoloration; 2—more severe, but not lasting; 3—moderate and more lasting; 4—medium and lasting; 5—moderately heavy; 6—heavy; 7—very heavy; 8—nearly destroyed; 9—destroyed; 10—completely destroyed. Each treatment had three repetitions with 2 plants each.

### 4.5. Gas Chromatography with Flame Ionisation Detector and Mass Spectrometry of Selected EOs and PE

Analysis of PE and EOs was carried out on an Agilent 8890 GC System gas chromatograph with two independent channels, DB-1MS capillary quartz columns 60 m long, 0.250 mm in diameter, stationary phase film thickness 0.25 µm, using a mass spectrometric detector (MSD) Agilent 5977B and flame ionization detector (FID) manufactured by Agilent Technologies, Inc. (Santa Clara, CA, USA) according to the method [115] with modifications.

For the analysis of EOs, 1% solutions in methanol were prepared and the solution was injected into the device in an amount of 0.2 µL per MSD channel and 0.5 µL per FID channel. For PE analysis, a sample of the extract was placed in a vial and heated at 120 °C for 5 min. Using a gas syringe, 2500 µL of an equilibrium gas–vapor mixture was taken from the vial and injected into the device through a channel connected to the MSD.

The temperature program was as follows: injector temperature 250 °C, initial isotherm 35 °C—2 min, heating 5 °C/min up to 140 °C, then 10 °C/min up to 250 °C, final isotherm 250 °C—5 min. Helium was used as the carrier gas at a rate of 1.3 mL/min on the MSD channel and 1.0 mL/min on the FID channel. Air 400 mL/min and hydrogen 30 mL/min were used as auxiliary gases for FID. Source temperature 230 °C MSD, quadrupole temperature 150 °C, scanning mode—total ion current, FID temperature 260 °C. The spectra were identified using the NIST spectrum library (National Institute of Standards and Technology, Gaithersburg, MD, USA). The analysis was repeated in quadruplicate and the results were represented as the mean area of peaks ± standard deviation.

### 4.6. Control Psg and Cff Artificial Infection Using EOs and PE

The experiments on the use of the analyzed substances against Psg and Cff against an artificial infection of soybean seeds and leaves were carried out during June–August 2022 in an experimental greenhouse. In all variants, the tests were carried out on soybean cv. Kasatka (the harvest year 2021, the weight of 1000 seeds = 122.8 g).

#### 4.6.1. Control Psg on Seeds

The creation of an artificial infection Psg on seeds was carried out according to the method [22]. Briefly, 72 h culture of Psg CFBP 2214 was resuspended in sterile 10 mM MgCl_2_ to ~10^4^ CFU/mL. Soybean seeds were sterilized in 75% ethanol, washed with an aqueous 50% solution of commercial bleach (sodium hypochlorite)/0.002% Tween 20 (*v*/*v*) for 8–10 min and distilled H_2_O until chlorine was removed, and left in a humid chamber for 2 h to swell. The swollen seeds were pierced with a sterile toothpick, transferred to a flask with a bacterial suspension, vacuum treated at –10^5^ Pa for 10 min, and dried to remove excess liquid.

The seeds were treated by immersion in an aqueous solution of (1) sterile water, (2) CCEO at a concentration of 0.5%, and (3) LBEE at a concentration of 13% for 10 min. After, the seeds were dried on paper towels to remove excess moisture. Thiram was used as a seed treater. Treatment with a standard seed treater was carried out at a drug consumption rate of 7 L/t and a working solution consumption of 8 L/t according to the pesticide registration data in the Russian Federation. To do this, 25 g of seeds and 200 μL of the treatment solution were placed in portions in a 50 mL tube (Eppendorf type) and thoroughly mixed in a microcentrifuge vortex for 2 min until the solution was completely absorbed into the seeds. The treated seeds were sown in a peat–perlite mixture (Veltorf, Velikie Luki, Russia) in 40-cell plastic transplant trays (cell volume 0.12 L, AgrofloraPack, Vologda, Russia). Plants were kept in a greenhouse at 28/22 °C (14-h day/10-h night) in natural sunlight and watered as needed. The treatments in each experiment were arranged in a complete randomization design. There were five replicates in each treatment with 40 seeds (1 tray per replicate).

#### 4.6.2. Control Psg on Leaves

The Psg artificial infection was created on vegetative plants according to the method [22] by infiltration of a bacterial suspension into a soybean leaf using an 1113 AirControl airbrush (JAS, Ninbo, China). Briefly, a bacterial suspension was prepared for seed infection with the addition of surfactant Silwet Gold (Chemtura, Philadelphia, PA, USA) to a concentration of 0.01% (*w*/*w*). Trifoliate leaves of plants were infected at stage V2 by treatment with an average dose of 5 mL of a suspension of bacteria at a concentration of 109 CFU/mL per trifoliate leaf. Plants were grown in plastic pots with a volume of 0.5 L as in paragraph 4.6.1. Two days before and 24 h after inoculation, relative humidity was maintained at ~95% at a constant temperature of 27 °C. The treatment of vegetative plants with the studied substances was carried out using 35-day-old soybean plants 2 h after bacterial inoculation at a consumption rate of the working solution of preparations of ~5 mL per plant (until all leaves were completely wetted) using a manual spray gun (with a droplet size of ~300 µm).

The design of the experiment included the use of (1) sterile water, (2) CCEO at a concentration of 0.5%, (3) LBEE at a concentration of 13%, and 4) foliar reference pesticide with bactericidal action Kocide 2000, WDG ((copper hydroxide 350 g/kg) Corteva Agriscience, Indianapolis, IN, USA). The reference foliar pesticide was used with a working solution concentration of 0.6% according to the preparation (according to the manufacturer’s recommendation).

The disease rate was recorded as the percentage of plants that showed leaf symptoms. The assessment of the development of the disease, in terms of infection of adult plants, was carried out on the 12th day after infection using the LeafDoctor application (https://www.quantitative-plant.org/software/leaf-doctor, accessed on 21 July 2022), installed on an iPhone SE 2. All leaves from all plants were individually photographed and analyzed by moving the threshold slider until only symptomatic tissues were converted to blue and calculating the percentage of diseased tissue as recommended by the developer [116]. In the seed treatment experiment, similar calculations were made, but after reaching stage V3 (35 days after sowing).

#### 4.6.3. Control Cff on Seeds

For the artificial infection of seeds, we used the method of damaging the hilum (hilum) with a sterile seed needle described in [117] with modifications. To do this, each seed was damaged by piercing the hilum with a sterile needle and soaked in a bacterial suspension; the mixture was then placed in a vacuum and dried on paper towels.

Soybean seeds were treated by immersion in an aqueous solution of (1) sterile water, (2) OEO at a concentration of 0.5%, (3) LBEE at a concentration of 13% for 10 min, and dried on paper napkins remove excess moisture. Thiram was used as the reference seed treater by analogy with point 4.6.1. Further actions with plants and growing conditions were similar to Section 4.6.1.

At 15, 18, 21, 24, 27, and 31 days after sowing, the severity of bacterial wilt disease of each plant was assessed with disease scores ranging from 0 to 5, where 0 = no symptoms of wilt; 1 = wilting on one of the primary leaves; 2 = wilting on both primary leaves, but not on the first trifoliate; 3 = withering of the first trifoliate leaf; 4 = death of the seedling after the development of primary leaves, and 5 = no germination or complete wilting and loss of turgor (in adult plants) of the soybean scale we adapted in a previous study described in [118]. AUPDC (Area Under Progress Disease Curve) was calculated according to the method [23] using the above scale in MS Excel 2007.

#### 4.6.4. Control Cff on Leaves

The Cff infection of vegetative soybean plants and the method for calculating plant disease were similar to that of Section 4.6.2. The design of the experiment included the use of (1) sterile water, (2) OEO at a concentration of 0.5%, (3) LBEE at a concentration of 13%, and (4) foliar reference pesticide with bactericidal action Kocide 2000, WDG. The calculation of the incidence rate, recurrence, and plant growing conditions was similar to Section 4.6.2.

### 4.7. Statistical Analysis

For the EOs and PEs disc inhibition zone experiment, the means were analyzed by one-way analysis of variance (ANOVA) followed by a Tukey post hoc multiple comparison test using the Statistica 12.0 software package (StatSoft, TIBCO, Palo Alto, CA, USA). Results were expressed as mean (M) ± standard deviation (SD). *p* values < 0.05 were considered significant.

For the rest of the experiments, including the determination of MIC and MBC, phytotoxicity, and control against an artificial infection on soybean, data analysis was carried out using the analysis of variance method using Statistica 12.0 (StatSoft, TIBCO, Palo Alto, CA, USA), comparing the average values by the criterion Duncan. The percentage data were converted to arcsine before processing. Graphs were created using GraphPad Prism 9.2.0. (GraphPad Software Inc., San Diego, CL, USA).

## 5. Conclusions

The results of this study showed that of 19 essential oils and 19 plant extracts, high antibacterial activity is displayed by the essential oils of Chinese cinnamon (against Psg), oregano (against Cff) and the ethanol extract of leather bergenia (for both bacteria). Moreover, the experiment with plants on an artificial infection of two bacterial diseases showed that these substances in non-phytotoxic concentrations can reduce the harmful effect of Psg and Cff when treating both infected seeds and leaves. These results are intriguing as they suggest that EOs and PEs could potentially be used as alternatives to traditional chemical pesticides and antibiotics in the control of soybean bacterial blight, tan spot, and wilt. However, before any promising application of the studied EOs and PEs as natural (botanical pesticides) to control phytopathogenic bacteria, it is necessary to evaluate potential side effects on non-target organisms, select an effective formulation, and conduct field studies in commercial crop production.

## Figures and Tables

**Table 1:** MIC (minimal inhibitory concentration) and MBC (minimal bactericidal concentration, calculated for each substance/bacteria pair) values. MIC values were calculated by counting the bacterium concentration (CFU/mL) after culturing in a liquid medium with each substance. ND—not determined (MIC and MBC values were determined only for EO/PE that showed activity on disc diffusion method). Solvents (for plant extracts): ETH—96% ethanol, W—water.

EO/PE/Antibiotic/Reference Pesticide	Bacteria (Strain)			
	PSG (G2)		CFF (F-125-1)	
	MIC	MBC	MIC	MBC
gentamicin	50	80	50	100
thiram	200	360	400	720
Essential oils				
*Cinnamomum aromaticum*	200	280	400	560
*Thymus vulgaris*	1200	1440	400	720
*Origanum vulgare*	1600	3200	200	280
*Mentha longifolia*	1600	2560	1200	1520
*Mentha piperita*	1600	3200	1600	3200
*Syzygium aromaticum*	1200	1600	ND	ND
*Lavandula angustifolia*	1600	3200	1600	3200
*Achillea millefolium*	1600	2880	1600	3200
*Allium sativum*	ND	ND	>3200	>3200
*Oleum calami*	>3200	>3200	ND	ND
*Citrus aurantiifolia*	>3200	>3200	ND	ND
*Elettaria cardamomum*	>3200	>3200	ND	ND
*Citrus reticulata*	>3200	>3200	ND	ND
*Pimpinella anisum*	>3200	>3200	ND	ND
*Foeniculum vulgare*	>3200	>3200	ND	ND
*Sālvia officinālis*	ND	ND	>3200	>3200
Extracts				
*Bergenia crassifolia* ETH	1000	4000	2500	5000
*Melissa officinalis* ETH	2500	5000	ND	ND
*Capsicum annuum* ETH	5000	9000	ND	ND
*Sambucus nigra* ETH	5000	10,000	ND	ND
*Phytolacca americana* ETH	5000	10,000	ND	ND
*Capsicum annuum* W	10,000	50,000	5000	9000
*Galega officinalis* W	10,000	50,000	10,000	50,000
*Artemisia absinthium* ETH	10,000	50,000	ND	ND
*Phellodendron amurense* ETH	50,000	100,000	50,000	>100,000
*Rosa pendulina* ETH	100,000	>100,000	ND	ND
*Chelidonium majus* ETH	ND	ND	5000	10,000
*Morus nigra* ETH	ND	ND	50,000	>100,000
*Spiraea salicifolia* W	ND	ND	100,000	>100,000
*Oleum calami* ETH	ND	ND	100,000	>100,000

**Table 2:** Chemical composition of leather bergenia ethanolic extract, Chinese cinnamon, and oregano essential oils. ^a^ MSD—Mass Spectrometric Detector; ^b^ FID—Flame Ionization Detector. The results represent the mean ± standard deviation of three different samples of each species analyzed individually in triplicate.

Retention Time (min)		Compound	Area ± SD, %		
^a^ MSD	^b^ FID		LBEE	CCEO	OEO
9.5 ± 0.03	-	Acetic acid	27.91 ± 0.12	-	-
11.54 ± 0.0	-	Propanoic acid	0.93 ± 0.09	-	-
14.21 ± 0.01	-	2,3-Butanediol	0.63 ± 0.02	-	-
15.93 ± 0.0	-	Isovaleric acid	1.04 ± 0.06	-	-
16.26 ± 0.0	-	2-Methylbutyric acid	0.13 ± 0.01	-	-
19.39 ± 0.0	18.48 ± 0.02	3-Thujene	-	-	0.33 ± 0.018
19.67 ± 0.0	18.75 ± 0.0	α-Pinene	-	-	1.65 ± 0.015
19.68 ± 0.01	18.76 ± 0.0	Benzaldehyde	-	0.08 ± 0.002	-
20.05 ± 0.0	19.15 ± 0.01	α-Fenchene	-	-	0.06 ± 0.002
20.12 ± 0.0	19.22 ± 0.0	Camphene	-	0.08 ± 0.01	0.45 ± 0.013
20.33 ± 0.02	-	Hexanoic acid	6.91 ± 0.14	-	-
20.97 ± 0.01	20.12 ± 0.0	β-Pinene	-	-	0.46 ± 0.004
21.16 ± 0.03	20.36 ± 0.0	β-Myrcene	-	-	0.84 ± 0.009
21.52 ± 0.02	20.77 ± 0.0	2,6-Dimethyl 2,6-octadiene	-	-	0.03 ± 0.0
21.67 ± 0.03	20.91 ± 0.01	Pseudolimonen	-	-	0.07 ± 0.004
22.07 ± 0.01	21.33 ± 0.0	α-Terpinene	-	-	0.53 ± 0.01
22.16 ± 0.0	21.41 ± 0.0	Cymene	-	-	19.85 ± 0.36
22.35 ± 0.0	21.61 ± 0.0	p-1-Menthene	-	-	0.035 ± 0.014
22.46 ± 0.0	21.73 ± 0.0	Eucalyptol, Limonene	-	-	0.28 ± 0.003
22.50 ± 0.03	-	5-Methyl-3-methylenedihydro-2(3H)-furanone	20.32 ± 0.71	-	-
22.53 ± 0.0	21.80 ± 0.0	Limonene	-	0.03 ± 0.001	-
23.27 ± 0.0	22.61 ± 0.02	γ-Terpinene	-	-	4.85 ± 0.002
23.92 ± 0.0	23.30 ± 0.0	D-Fenchone	-	-	0.04 ± 0.0
24.17 ± 0.01	23.61 ± 0.0	Linalool	-	-	2.53 ± 0.045
24.32 ± 0.03	23.86 ± 0.0	Phenylethyl Alcohol	0.29 ± 0.001	0.15 ± 0.007	-
25.36 ± 0.01	24.87 ± 0.0	d-Camphor	-	-	0.072 ± 0.009
25.43 ± 0.0	24.96 ± 0.0	3-Phenylpropanal	-	0.10 ± 0.026	-
25.63 ± 0.0	-	l-Menthone	1.69 ± 0.08	-	-
25.86 ± 0.0	-	Isomenthone	1.25 ± 0.01	-	-
25.87 ± 0.0	25.42 ± 0.01	4-Phenylbutanal	-	0.09 ± 0.01	-
26.04 ± 0.02	25.61 ± 0.0	endo-Borneol	-	0.1 ± 0.032	0.10 ± 0.03
26.06 ± 0.0	-	Isoborneol	0.46 ± 0.003	-	-
26.29 ± 0.01	25.9 ± 0.01	Terpinen-4-ol	-	-	0.04 ± 0.001
26.31 ± 0.01	-	trans-Sabinene hydrate	0.53 ± 0.006	-	-
26.47 ± 0.0	-	Methyl salicylate	5.18 ± 0.08	-	-
26.97 ± 0.01	-	2-Hydroxycineole	1.16 ± 0.08	-	-
27.27 ± 0.0	26.91 ± 0.01	2-Anisaldehyde	-	0.31 ± 0.037	-
27.49 ± 0.01	-	Pulegone	4.46 ± 0.0	-	-
27.58 ± 0.01	27.32 ± 0.01	Thymol methyl ether	-	-	0.15 ± 0.08
27.76 ± 0.02	-	Piperitone	0.66 ± 0.07	-	-
27.88 ± 0.0	27.53 ± 0.0	(E)-Cinnamaldehyde	-	84.25 ± 3.452	-
28.34 ± 0.0	28.13 ± 0.0	Thymol	3.33 ± 0.02	-	3.51 ± 0.055
28.57 ± 0.0	28.37 ± 0.0	Carvacrol	0.45 ± 0.01	-	62.37 ± 0.01
29.60 ± 0.0	-	Eugenol	10.89 ± 0.61	-	-
30.35 ± 0.0	30.24 ± 0.0	Cinnamaldehyde dimethyl acetal	-	3.36 ± 0.011	-
30.54 ± 0.0	30.43 ± 0.0	Copaene	-	0.38 ± 0.007	-
30.76 ± 0.01	30.71 ± 0.0	Coumarin	-	0.38 ± 0.001	-
30.90 ± 0.0	30.83 ± 0.0	Cinnamyl acetate	-	2.34 ± 0.052	-
31.22 ± 0.0	31.18 ± 0.01	Caryophyllene	-	-	0.6 ± 0.002
31.84 ± 0.01	31.82 ± 0.0	Curcumene	-	0.15 ± 0.04	-
31.94 ± 0.02	31.93 ± 0.01	γ-Amorphene	-	0.17 ± 0.0	-
32.02 ± 0.0	-	3,5-Di-tert-butylphenol	1.6 ± 0.03	-	-
32.09 ± 0.0	32.08 ± 0.01	o-Methoxycinnamaldehyde	-	6.93 ± 0.024	-
32.26 ± 0.0	32.25 ± 0.01	α-Muurolene	-	0.34 ± 0.01	-
33.28 ± 0.01	-	2,2,4-Trimethyl-1,3-pentanediol diisobutyrate	0.35 ± 0.01	-	-
33.32 ± 0.0	33.39 ± 0.01	α-Farnesene	-	-	0.07 ± 0.002
33.35 ± 0.01	33.42 ± 0.0	Methoxycinnamaldehyde dimethyl acetal	-	0.4 ± 0.026	-
33.38 ± 0.01	33.46 ± 0.0	Caryophylene oxide	-	-	0.63 ± 0.03
-	-	**Total identified**	90.17	99.64	99.55

**Table 3:** Plant material is used for the preparation of essential oils and extracts.

Common Name	Latin Name	Family	Part of the Plant	EO or PE	References
Sweet-flag	*Oleum calami* L.	*Acoraceae*	rhizomes	EO, PE	[85,86]
Common yarrow	*Achillea millefolium* L.	*Asteraceae*	inflorescences	EO	[46]
Fennel	*Foeniculum vulgare* Mill.	*Apiaceae*	fruit	EO	[87]
Anise	*Pimpinella anisum* L.	*Apiaceae*	fruit	EO	[88]
Tansy	*Tanacetum vulgare* L.	*Asteraceae*	leaves and inflorescences	EO	[87]
Garlic	*Allium sativum* L.	*Amaryllidaceae*	bulbs	EO, PE	[89,90]
Thyme	*Thymus vulgaris* L.	*Lamiaceae*	leaves and inflorescences	EO	[37]
Oregano	*Origanum vulgare* L.	*Lamiaceae*	leaves and stems	EO	[52]
Peppermint	*Mentha piperita* L.	*Lamiaceae*	flowers, leaves, stems	EO	[87]
Horse mint	*Mentha longifolia* (L.) Huds.	*Lamiaceae*	flowers, leaves, stems	EO	[91]
English lavender	*Lavandula angustifolia* Mill.	*Lamiaceae*	leaves and inflorescences	EO	[92]
Rosemary	*Rosmarinus officinalis* L.	*Lamiaceae*	leaves and inflorescences	EO	[87]
Sage	*Salvia officinālis* L.	*Lamiaceae*	leaves and flowers	EO	[87]
Chinese cinnamon	*Cinnamomum aromaticum* (L.) Presl	*Lauraceae*	leaves	EO	[54]
Clove	*Syzygium aromaticum* (L.) Merr. & Perry	*Myrtaceae*	flowers, leaves, stems	EO	[85]
Key lime	*Citrus aurantiifolia* (Christm.) Swingle	*Rutaceae*	fruit peel	EO	[85]
Mandarin orange	*Citrus reticulata* Blanco	*Rutaceae*	fruit peel	EO	[87]
Common rue	*Ruta graveolens* L.	*Rutaceae*	leaves and stems	EO	[93]
Green cardamom	*Elettaria cardamomum* (L.) Maton	*Zingiberaceae*	fruit	EO	[87]
Caraway	*Carum carvi* L.	*Apiaceae*	fruit	PE	[36]
Wormwood	*Artemisia absinthium* L.	*Asteraceae*	leaves and stems	PE	[94]
Elderberry	*Sambucus nigra* L.	*Caprifoliaceae*	leaves	PE	[95]
Galega	*Galega officinalis* L.	*Fabaceae*	leaves and stems	PE	[96]
Common oak	*Quercus robur* L.	*Fagaceae*	leaves	PE	[97]
Lemon balm	*Melissa officinalis* L.	*Lamiaceae*	leaves and stems	PE	[98]
Black mulberry	*Morus nigra* L.	*Moraceae*	leaves	PE	[99]
Greater celandine	*Chelidonium majus* L.	*Papaveraceae*	leaves and stems	PE	[100]
Plume poppy	*Macleaya cordata* (Willd.) R. Br.	*Papaveraceae*	leaves and stems	PE	[97]
Pokeweed	*Phytolacca americana* L.	*Phytolaccaceae*	leaves and stems	PE	[100]
Giant knotweed	*Reynoutria sachalinensis* (F.Schmidt) Nakai,	*Polygonaceae*	leaves and stems	PE	[101]
Amur cork tree	*Phellodendron amurense* Rupr. (1857)	*Rutaceae*	leaves	PE	[102]
Alpine rose	*Rosa pendulina* L.	*Rosaceae*	leaves	PE	[103]
Bridewort	*Spiraea salicifolia* L.	*Rosaceae*	leaves and stems	PE	[104]
Leather bergenia	*Bergenia crassifolia* (L.) Fritsch	*Saxifragaceae*	rhizomes	PE	[105]
Cayenne pepper	*Capsicum annuum* L.	*Solanaceae*	fruit	PE	[106]
Manchurian walnut	*Juglans mandshurica* Maxim.	*Juglandaceae*	leaves	PE	[107]

## Data Availability

Not applicable.

## Supplementary Materials

The following supporting information can be downloaded at https://www.mdpi.com/article/10.3390/plants11212989/s1, Figure S1. Phytotoxicity on seedlings by soaking seeds in an aqueous solution (A) and treating soybean leaves (B) with various concentrations of Chinese cinnamon EO. Shown are 2 typical seeds (8 DAT) and 1 trifoliate leaf (7 DAT) from each sample before counting; Figure S2. Chromatographic profile of ethanolic extract of leather bergenia using a mass spectrometric detector; Figure S3. Chromatographic profile of Chinese cinnamon EO using MSD (A) and FID (B); Figure S4. Chromatographic profile of oregano EO using MSD (A) and FID (B); Figure S5. Symptoms of Psg and Cff on soybean leaves 12 days after infection of the leaves with an airbrush. Table S1. The activity of essential oils, plant extracts, antibiotics, and reference pesticide against Pseudomonas savastanoi pv. glycinea and Curtobacterium *flaccumfaciens* pv. *flaccumfaciens* strains were measured as growth inhibition zones in the agar-well diffusion method.

## Abbreviations

LBEE	Leather Bergenia Ethanolic Extract
CCEO	Chinese Cinnamon Essential Oil
OEO	Oregano Essential Oil
a.i.	active ingredient
WSC	Water Suspension Concentrate
WDG	Water-Dispersible Granules
PGPR	Plant Growth-Promoting Rhizobacteria
MSD	Mass Spectrometric Detector
FID	Flame Ionization Detector
