# Supplementary material for: Using of Essential Oils and Plant Extracts against Pseudomonas savastanoi pv. glycinea and Curtobacterium flaccumfaciens pv. flaccumfaciens on Soybean
Source: Plants (Basel). 2022 Nov 5;11(21):2989. doi: 10.3390/plants11212989 (PMC9655289; doi:10.3390/plants11212989)
Supplement: Supplementary file 1 [file plants-11-02989-s001.zip › plants-1978933-supplementary.pdf]

plants

MDPI

Article

Using of Essential Oils and Plant Extracts against *Pseudomonas savastanoi* pv. *glycinea* and *Curtobacterium flaccumfaciens* pv. *flaccumfaciens* on Soybean

Rashit Tarakanov, Fevzi S.-U. Dzhalilov

Department of Plant Protection, Russian State Agrarian University—Moscow Timiryazev Agricultural Academy, Timiryazevskaya Str. 49, 127434 Moscow, Russia
* Correspondence: tarakanov.rashit@mail.ru (R.T.); labzara@mail.ru (F.S.-U.D.)

Supplementary material.

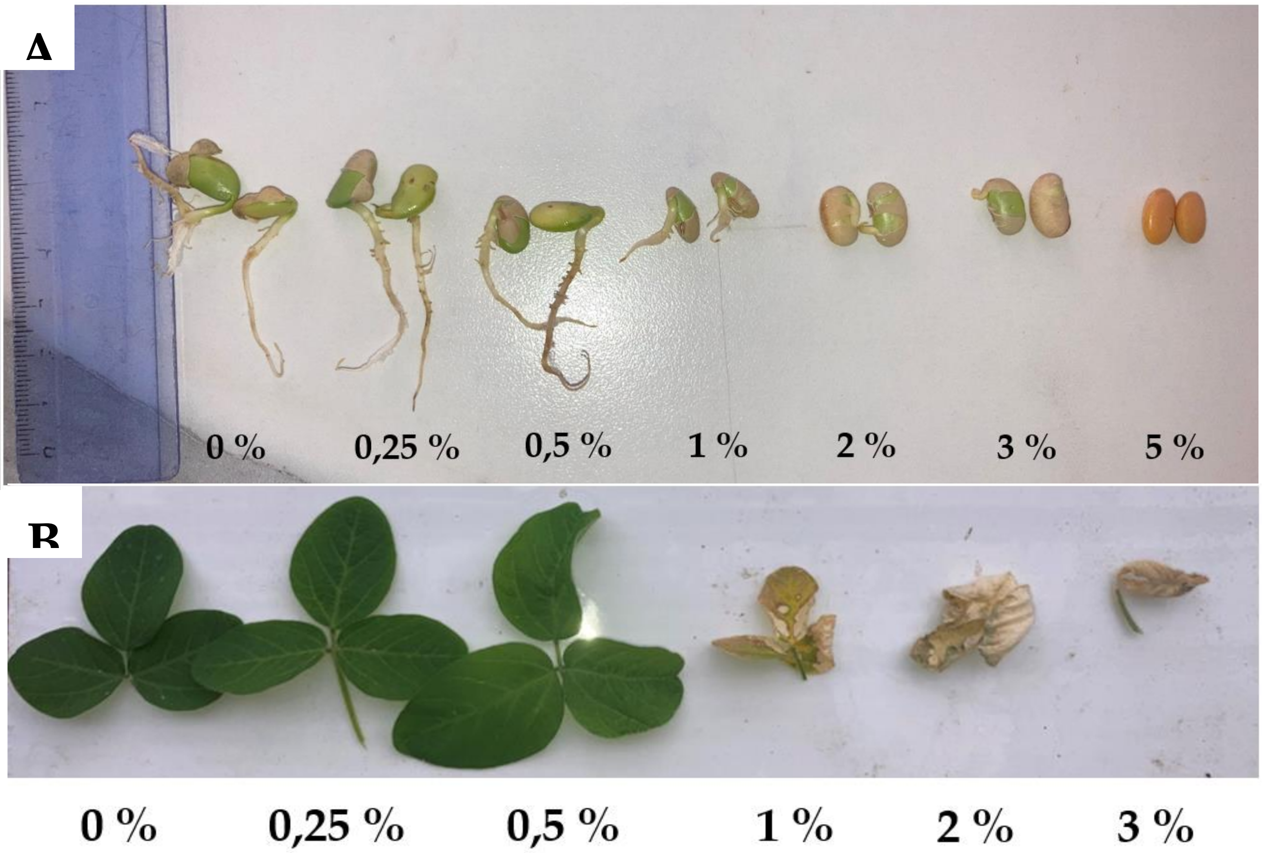

Supplementary Figure S1. Phytotoxicity on seedlings by soaking seeds in an aqueous solution (A) and treating soybean leaves (B) with various concentrations of chinese cinnamon EO. Shown are 2 typical seeds (8 DAT) and 1 trifoliate leaf (7 DAT) from each sample before counting.

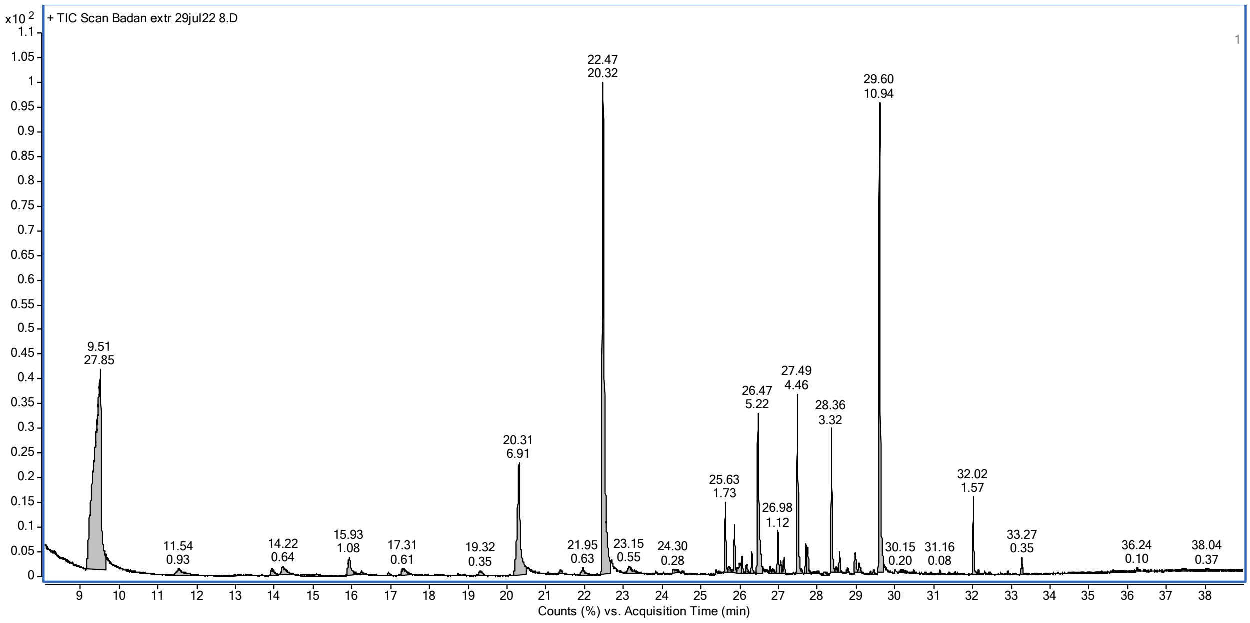

Supplementary Figure S2. Chromatographic profile of ethanolic extract of leather bergenia using a mass spectrometric detector.

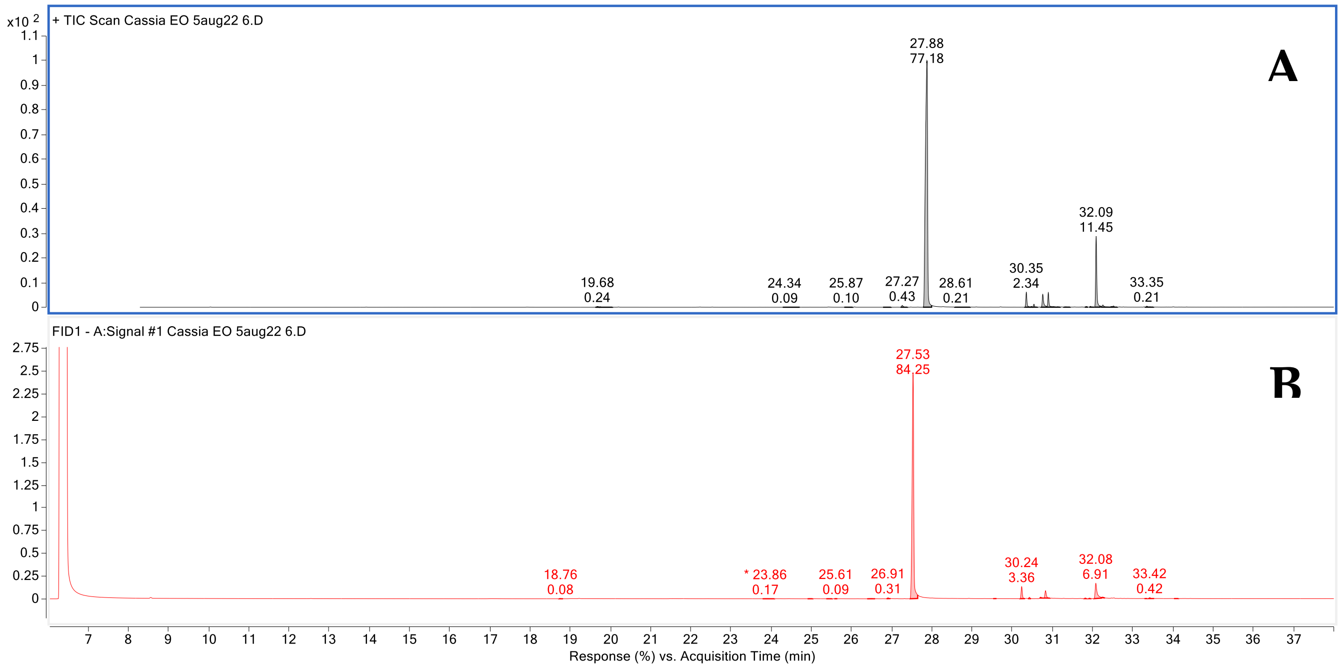

Supplementary Figure S3. Chromatographic profile of chinese cinnamon EO using MSD (A) and FID (B).

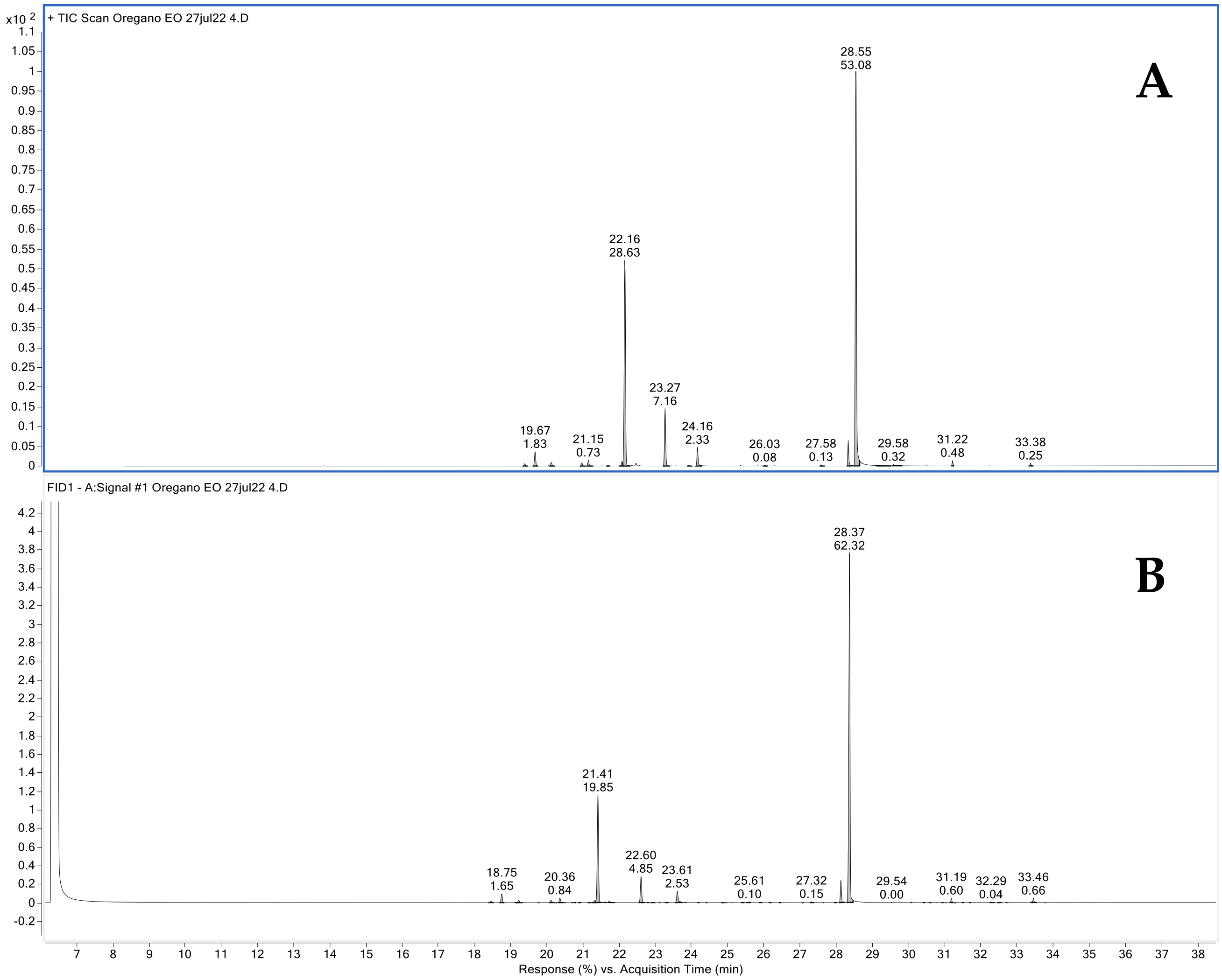

Supplementary Figure S4. Chromatographic profile of oregano EO using MSD (A) and FID (B).

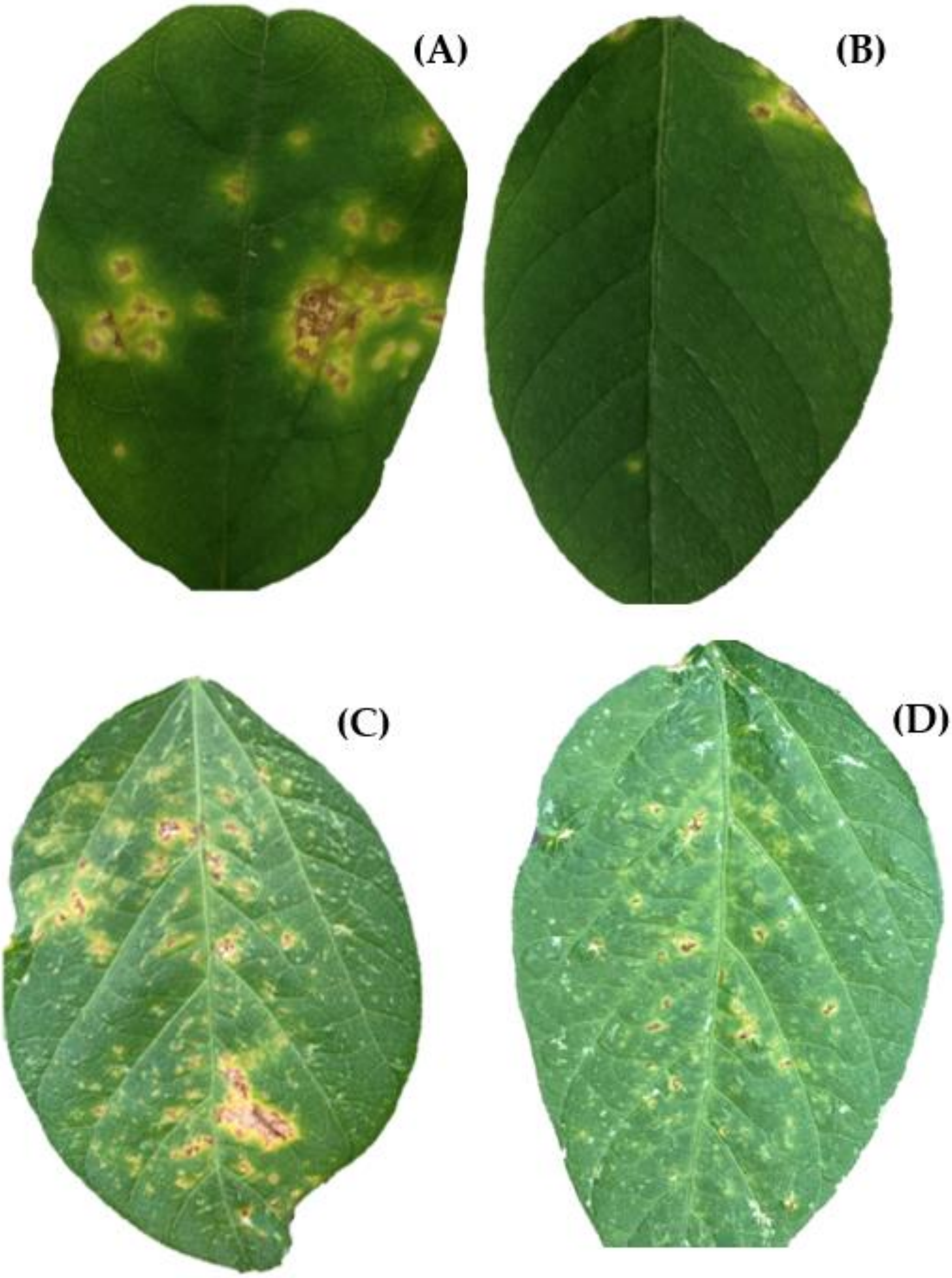

Supplementary Figure S5. Symptoms of Psg and Cff on soybean leaves 12 days after inoculation with an airbrush. (A) positive control (water treatment of infected leaves; Psg infection); (B) treatment with CCEO (Psg infection); (C) positive control (water treatment of infected leaves; Cff infection); (D) treatment with OEO (Cff infection). Typical leaves from the samples are presented.

Supplementary Table S1. The activity of essential oils, plant extracts, antibiotics, and reference pesticides against *Pseudomonas savastanoi* pv. *glycinea* and *Curtobacterium flaccumfaciens* pv. *flaccumfaciens* strains were measured as growth inhibition zones in the agar-well diffusion method. M – mean; SD – standard deviation; a-e —the same letter is not significantly different (ANOVA. α = 0.05; Tukey's test. α= 0.05). Solvents for extracts: ETH – 96% ethanol, W- water.

**Strain** / **EO/PE/ antibiotic/ reference pesticide**		**Bacteria growth inhibition zone M±SD, mm**					
		**Psg strains**			**Cff strains**		
		CFBP 2214	G2	G17	CFBP 3418	F-125-1	F-30-1
gentamicin		22.7±0.5^{e}	21.7±0.5^{e}	22.7±0.5^{e}	20.7±0.5^{e}	20.7±0.5^{e}	20.3±0.5^{e}
thiram		4.3±0.5^{c}	4.3±0.5^{c}	4.3±0.5^{c}	6.3±0.5^{d}	6.3±0.5^{d}	6.3±0.5^{d}
**Essential oils**							
Cinnamomum aromaticum		9.7±0.5^{d}	9.0±0^{d}	9.7±0.5^{d}	2.0±0^{b}	2.3±0.5^{b}	2.0±0^{b}
Thymus vulgaris		2.7±0.5^{b}	2.7±0.5^{b}	2.7±0.5^{b}	5.7±0.5^{c}	5.7±0.5^{c}	5.7±0.5^{c}
Origanum vulgare		2.0±0.8^{b}	2.0±0.8^{b}	2.0±0.8^{b}	8.3±0.5^{cd}	8.3±0.5^{cd}	8.0±0^{cd}
Mentha piperita		1.3±0.5^{b}	1.3±0.5^{b}	1.3±0.5^{b}	4.7±0.5^{b}	4.0±0.8^{b}	4.7±0.5^{b}
Allium sativum		0±0^{a}	0±0^{a}	0±0^{a}	3.7±0.5^{c}	3.7±0.5^{c}	3.7±0.5^{c}
Acorus calamus		5.7±0.5^{c}	6.0±0^{c}	5.7±0.5^{c}	0±0^{a}	0±0^{a}	0±0^{a}
Mentha longifolia		2.7±0.5^{b}	2.7±0.5^{b}	2.7±0.5^{b}	4.3±0.5^{b}	4.7±0.5^{b}	4.3±0.5^{b}
Citrus aurantiifolia		6.0±0^{d}	6.0±0^{d}	6.0±0^{d}	0±0^{a}	0±0^{a}	0±0^{a}
Syzygium aromaticum		9.3±0.5^{d}	9.0±0.5^{d}	9.3±0.5^{d}	0±0^{a}	0±0^{a}	0±0^{a}
Elettaria cardamomum		6.0±0.8^{c}	6.0±0.8^{c}	6.0±0.8^{c}	0±0^{a}	0±0^{a}	0±0^{a}
Citrus reticulata		6.3±0.5^{c}	6.0±0^{c}	6.3±0.5^{c}	0±0^{a}	0±0^{a}	0±0^{a}
Pimpinella anisum		5.3±0.5^{c}	5.3±0.5^{c}	5.3±0.5^{c}	0±0^{a}	0±0^{a}	0±0^{a}
Lavandula angustifolia		2.3±0.5^{b}	2.3±0.5^{b}	2.3±0.5^{b}	5.3±0.5^{c}	5.3±0.5^{c}	5.3±0.5^{d}
Ruta graveolens		0±0^{a}	0±0^{a}	0±0^{a}	0±0^{a}	0±0^{a}	0±0^{a}
Tanacetum vulgare		0±0^{a}	0±0^{a}	0±0^{a}	0±0^{a}	0±0^{a}	0±0^{a}
Rosmarinus officinalis		3.3±0.5^{b}	3.0±0.8^{b}	2.3±0.6^{b}	0±0^{a}	0±0^{a}	0±0^{a}
Achillea millefolium		3.0±0^{b}	4.0±0.8^{b}	2.7±0.6^{b}	3.3±0.5^{b}	3.0±1.0^{b}	3.0±0^{b}
Foeniculum vulgare		5.3±0.5^{d}	5.7±0.5^{d}	6.3±0.6^{d}	0±0^{a}	0±0^{a}	0±0^{a}
Salvia officinalis		0±0^{a}	0±0^{a}	0±0^{a}	4.3±0.5^{b}	4.0±0^{b}	4.3±0.5^{b}
**Extracts**							
Phellodendron amurense	ETH	2.0±0^{b}	1.7±0.5^{b}	1.7±0.5^{b}	1.7±0.5^{b}	1.7±0.5^{b}	1.7±0.5^{c}
	W	0±0^{a}	0±0^{a}	0±0^{a}	0±0^{a}	0±0^{a}	0±0^{a}
Bergenia crassifolia	ETH	5.0±0^{c}	5.3±0.5^{b}	5.3±0.5^{c}	6.0±0^{c}	6.0±0^{c}	6.3±0.5^{c}
	W	0±0^{a}	0±0^{a}	0±0^{a}	0±0^{a}	0±0^{a}	0±0^{a}
Capsicum annuum	ETH	1.7±0.9^{b}	1.3±0.5^{b}	1.3±0.5^{b}	0±0^{a}	0±0^{a}	0±0^{a}
	W	1.7±0.5^{b}	1.7±0.5^{b}	1.7±0.5^{b}	1.7±0.5^{b}	1.7±0.5^{b}	1.3±0.5^{c}
Galega officinalis	ETH	0±0^{a}	0±0^{a}	0±0^{a}	0±0^{a}	0±0^{a}	0±0^{a}
	W	1.3±0.5^{b}	1.3±0.5^{b}	1.3±0.5^{b}	1.7±0.5^{b}	1.3±0.5^{b}	1.7±0.5^{c}
Artemisia absinthium	ETH	0±0^{a}	0±0^{a}	0±0^{a}	0±0^{a}	0±0^{a}	0±0^{a}

	W	0±0[a]	0±0[a]	0±0[a]	0±0[a]	0±0[a]	0±0[a]
Rosa pendulina	ETH	0±0[a]	0±0[a]	0±0[a]	0±0[a]	0±0[a]	0±0[a]
	W	0±0[a]	0±0[a]	0±0[a]	0±0[a]	0±0[a]	0±0[a]
Phytolacca americana	ETH	0±0[a]	0±0[a]	0±0[a]	0±0[a]	0±0[a]	0±0[a]
	W	0±0[a]	0±0[a]	0±0[a]	0±0[a]	0±0[a]	0±0[a]
Chelidonium majus	ETH	0±0[a]	0±0[a]	0±0[a]	1.7±0.5[b]	1.3±0.5[b]	1.3±0.5[c]
	W	0±0[a]	0±0[a]	0±0[a]	0±0[a]	0±0[a]	0±0[a]
Morus nigra	ETH	0±0[a]	0±0[a]	0±0[a]	1.3±0.5[b]	1.3±0.5[b]	1.7±0.5[c]
	W	0±0[a]	0±0[a]	0±0[a]	0±0[a]	0±0[a]	0±0[a]
Spiraea salicifolia	ETH	0±0[a]	0±0[a]	0±0[a]	0±0[a]	0±0[a]	0±0[a]
	W	0±0[a]	0±0[a]	0±0[a]	1.3±0.5[b]	1.3±0.5[b]	2.0±0[c]
Oleum calami	ETH	0±0[a]	0±0[a]	0±0[a]	1.3±0.5[b]	1.3±0.5[b]	2.3±0.5[b]
	W	0±0[a]	0±0[a]	0±0[a]	0±0	0±0[a]	0±0[a]
Melissa officinalis	ETH	1.7±0.5[b]	1.3±0.5[b]	1.3±0.5[b]	0±0[a]	0±0[a]	0±0[a]
	W	0±0[a]	0±0[a]	0±0[a]	0±0[a]	0±0[a]	0±0[a]
Sambucus nigra	ETH	2.0±0[b]	2.0±0[b]	2.0±0.6[b]	0±0[a]	0±0[a]	0±0[a]
	W	0±0[a]	0±0[a]	0±0[a]	0±0[a]	0±0[a]	0±0[a]
Quercus robur	ETH	0±0[a]	0±0[a]	0±0[a]	0±0[a]	0±0[a]	0±0[a]
	W	0±0[a]	0±0[a]	0±0[a]	0±0[a]	0±0[a]	0±0[a]
Reynoutria sachalinensis	ETH	0±0[a]	0±0[a]	0±0[a]	0±0[a]	0±0[a]	0±0[a]
	W	0±0[a]	0±0[a]	0±0[a]	0±0[a]	0±0[a]	0±0[a]
Tanacetum vulgare	ETH	0±0[a]	0±0[a]	0±0[a]	0±0[a]	0±0[a]	0±0[a]
	W	0±0[a]	0±0[a]	0±0[a]	0±0[a]	0±0[a]	0±0[a]
Carum carvi	ETH	0±0[a]	0±0[a]	0±0[a]	0±0[a]	0±0[a]	0±0[a]
	W	0±0[a]	0±0[a]	0±0[a]	0±0[a]	0±0[a]	0±0[a]
Juglans mandshurica	ETH	0±0[a]	0±0[a]	0±0[a]	0±0[a]	0±0[a]	0±0[a]
	W	0±0[a]	0±0[a]	0±0[a]	0±0[a]	0±0[a]	0±0[a]
Macleaya cordata	ETH	0±0[a]	0±0[a]	0±0[a]	0±0[a]	0±0[a]	0±0[a]
	W	0±0[a]	0±0[a]	0±0[a]	0±0[a]	0±0[a]	0±0[a]
